# Retrospective study of 149 cases of salivary gland carcinoma in a Spanish hospital population

**DOI:** 10.4317/medoral.21419

**Published:** 2017-02-04

**Authors:** Lucía Collazo-Fernández, Julián Campo-Trapero, Jorge Cano-Sánchez, Rosa García-Martín, Claudio Ballestín-Carcavilla

**Affiliations:** 1 DDS, MSD, Doctorate student. School of Dentistry, Complutense University of Madrid; 2 DDS, PhD, Lecturer. Department of Medicine and Orofacial Surgery, School of Dentistry, Complutense University of Madrid; 3 DDS, PhD, Private Activity. Specialist in Oral Surgery, UK; 4 Biologist. Department of Pathology of the 12 de Octubre Hospital of Madrid, Madrid; 5 MD, PhD, Physician. Department of Pathology of the 12 de Octubre Hospital of Madrid

## Abstract

**Background:**

The clinical and histological characteristics of salivary gland tumors vary widely, complicating their diagnosis and management, and major differences have been recorded in the distribution of histopathological diagnoses among different countries.

**Material and Methods:**

This retrospective study reviewed the demographic (age, sex) and clinicopathological (pathology diagnosis and localization) characteristics of cases diagnosed with primary SGC between June 1992 and May 2014 in the Pathology Department of the 12 de Octubre Hospital of Madrid. Diagnoses were recorded according to the 2005 WHO classification.

**Results:**

The study included 149 SCG patients, aged between 11 and 94 yrs, with mean age at onset of 55.56 yrs and peak incidence in the eighth decade of life. The male:female ratio was 1.01. The parotid gland was the most frequently involved (75.2%). The most frequent carcinoma was mucoepidermoid carcinoma (24.2%), followed by acinic cell carcinoma (15.4%).

**Conclusions:**

The demographic and histopathological characteristics of patients with salivary gland carcinomas in Spain, reported here for the first time, are broadly similar to those found in other countries.

**Key words:**Salivary gland carcinomas, descriptive, salivary glands, salivary gland tumors, head and neck cancer, oral cancer, Spain.

## Introduction

The human body contains three major salivary glands (parotid, submandibular, and sublingual) and hundreds of minor salivary glands distributed throughout the upper aerodigestive tract. Numerous types of tumor originate in the salivary glands, and their histopathology is considered to be more complex and diverse than that of tumors at other anatomic sites (1). Malignant neoplasms of salivary glands constitute 1% - 3% of all head and neck cancers and only 0.3% of all malignant neoplasms (2). The reported incidence of malignant and benign salivary gland neoplasms in different counties has ranged between 0.2 and 9.7 and between 1.1 and 2.9 per 100,000 inhabitants, respectively (3). The clinical and histological characteristics of salivary gland tumors vary widely, complicating their diagnosis and management (4), and major differences have been recorded in the distribution of histopathological diagnoses among different countries (5-7). This drawback was addressed by the WHO in 2005 with a reclassification of these neoplasms and the introduction of new entities, establishing 38 subtypes divided between epithelial and mesenchymal cases. Data on the distribution of subtypes and the characteristics of patients are useful to improve our understanding of this disease, but no such information has been published on the distribution of salivary gland carcinomas in Spain. The objective of this study was to determine the distribution of demographic and histopathological characteristics in patients with SGC in a Spanish hospital population.

## Material and Methods

Clinicopathological data of patients with primary malignant neoplasms of epithelial salivary gland origin were gathered from patients diagnosed in the Pathology Department of the 12 de Octubre Hospital of Madrid between June 1992 and May 2014. This type of tumor is treated by multiple specialists, including maxillofacial surgeons, otolaryngologists, dentists, plastic surgeons, and radiotherapists. Treatment was not considered in this study because the therapeutic approach varies among the specialties involved. A biopsy report was available for all diagnosed cases. The demographic and clinical characteristics reviewed include sex, age, localization, and histological type.

IBM SPSS Statistics 22 for Windows was used for the statistical analysis. Only definitive diagnoses were considered in the histopathological analysis and only primary lesions derived from salivary glands were included, excluding benign tumors, mesenchymal tumors, congenital anomalies, congenital cysts, chronic sialadenitis, sialolithiasis, vascular and lymphatic malformations, salivary or mixed cysts, granulomatous infections, and salivary gland invasions of skin carcinomas. All cases were recorded according to the 2005 WHO classification.

The current study was approved by Ethical Comitte of the 12 de Octubre Hospital (Nº CEIC 15/ 177).

## Results

A total of 149 cases with salivary gland carcinomas met the inclusion criteria between June 1992 and May 2014 and were reviewed in this study.

- Sex and age

The age of patients at diagnosis ranged between 11 and 94 yrs, with a mean (standard deviation [SD] age of 55.66 (20.0) yrs and a peak incidence in the eighth decade of life. Three cases (2%) were diagnosed in pediatric age (<16 yrs). The study included 75 males (50.3%) and 74 females (49.7%), a male:female (M:F) ratio of 1.01.

**Figure 1:**
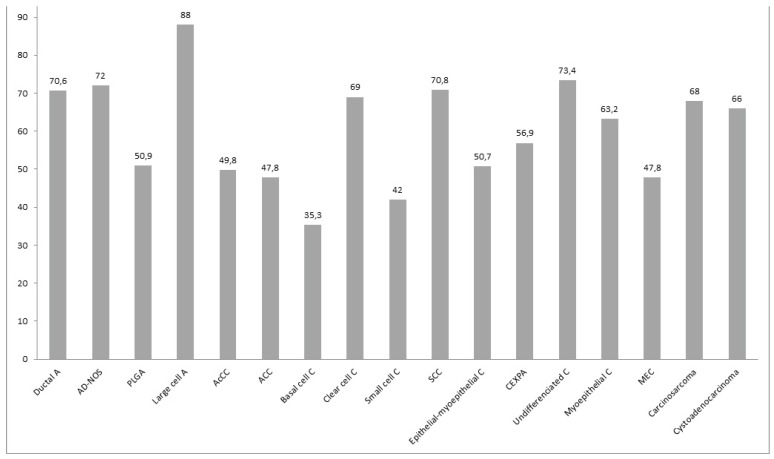
Mean age by histological type. AD-NOS= adenocarcinoma not otherwise specified, PLGA= polymorphous low grade adenocarcinoma, AcCC = acinic cell carcinoma, ACC= adenoid cystic carcinoma, SCC=squamous cell carcinoma, CEXPA = carcinoma ex-pleomorphic adenoma, C = undifferentiated carcinoma, and MEC= mucoepidermoid carcinoma.

The mean age at onset was lower in females (52.92 [21.3] yrs) than in males (58.43 [18.3] yrs). The age at diagnosis was 35.33 (14.01) yrs in patients with basal cell carcinoma, 47.8 (21.03) yrs in those with mucoepidermoid carcinoma (MEC), 47.8 (19.80) yrs in those with adenoid cystic carcinoma (ACC), 49.8 (18.63) yrs in those with acinic cell carcinoma (AcCC), 50.9 (18.56) yrs in those with carcinoma ex-pleomorphic adenoma, 50.9 (9.17) yrs In those with PLGA; 70.8 (14.01) yrs in those with squamous cell carcinoma (SCC), and 88 yrs in the patient with large cell carcinoma (Fig. 1).

- Localization

**Figure 2:**
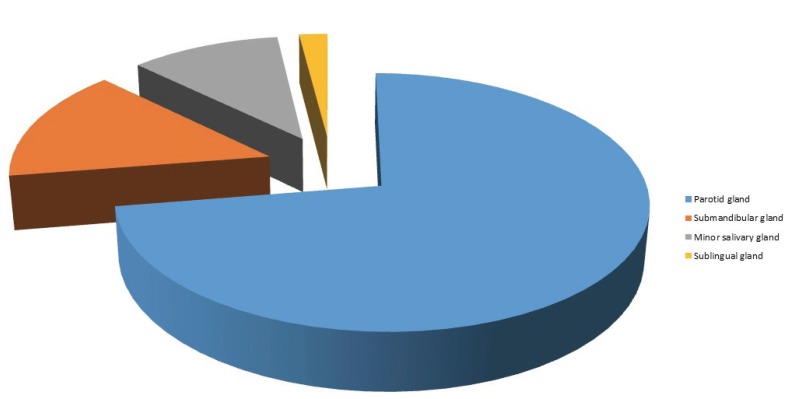
Distribution of salivary gland carcinomas by site (n= 149).

The most frequent tumor localization in our series was the parotid gland (n=108, 72.5%) followed by the submandibular gland (n= 22, 14.8%), minor salivary glands (n=16, 10.7%), and sublingual gland (n=3, 2%) (Fig. 2). Among minor salivary glands , the most frequent localization was the lip (n=5), followed by soft palate (n=4), buccal mucosa (n=3), and genian area (n=2).

- Histological diagnosis 

The most frequent carcinoma in our series was MEC (n=36, 24.2%), followed by AcCC (n=23; 15.4%), SCC (n=17; 11.4%), and ACC (n=16; 10.8%). MEC was equally prevalent in males and females (M:F=1), while SCC showed the highest male-female ratio (3.25). Myoepithelial carcinoma was the most frequent among females (M:F=0.33), followed by AcCC. Among males, SCC was the second most frequent, followed by ACC ( Table 1).

**Table 1:**
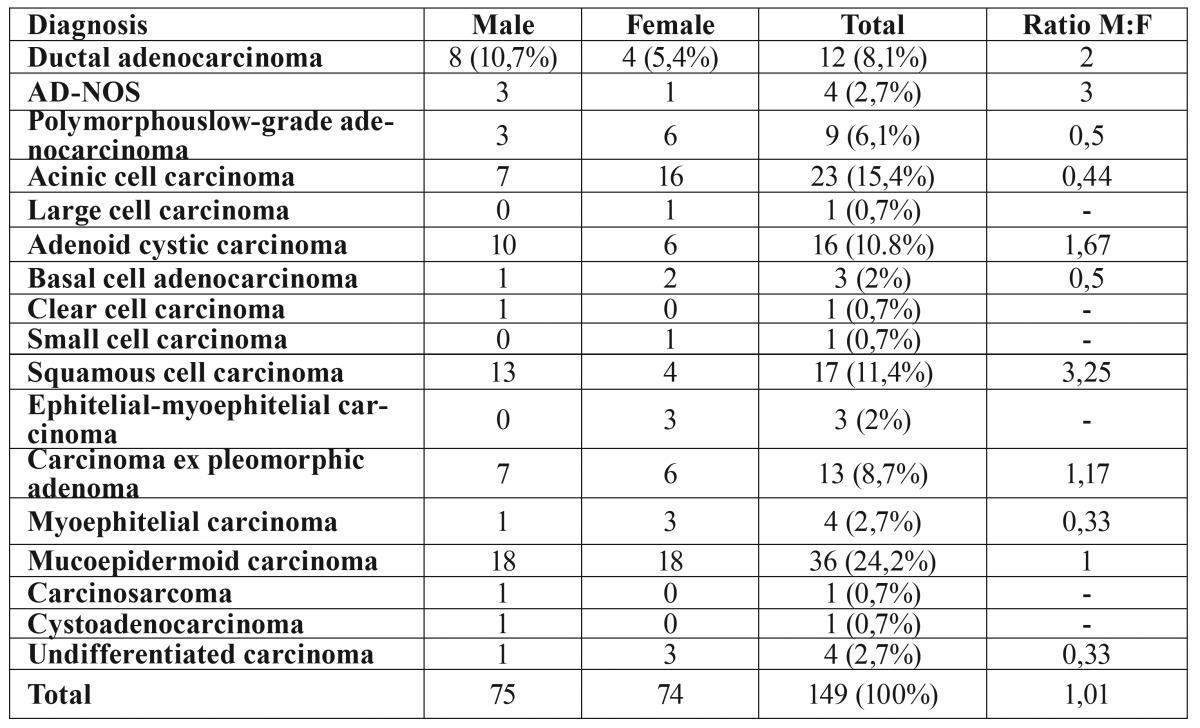
Classification of salivary gland carcinomas by diagnosis and sex.

PLGA (n=9) was only observed in minor salivary glands, where it was the most frequent type (56.3%). Among the 23 cases of AcCC, 22 (95.7%) were in the parotid gland, where it was the second most frequent type after MEC. In the submandibular gland, the most frequent type was MEC (n=6), followed by SCC (n=5) and ACC (n= 5) ( Table 2). Submandibular gland involvement was more frequent among males (n=18, 81.8%) than among females (n=4, 18.2%); while parotid gland involvement was more frequent among females (n=61; 56.5%) than among males (n=47; 43.5%). There was no gender difference in minor salivary glands involvement (50%).

**Table 2:**
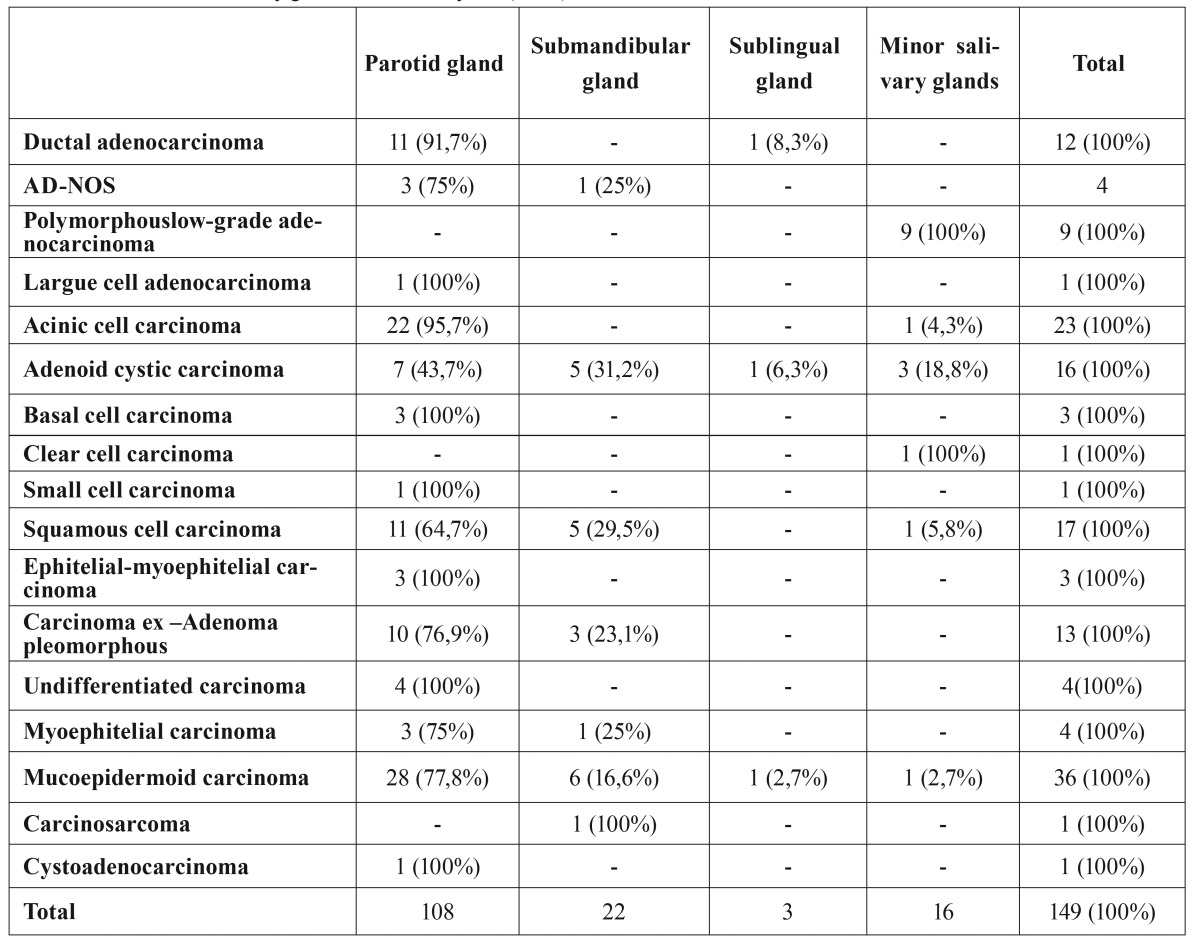
Distribution of salivary gland carcinomas by site (n= 49).

## Discussion

This study reports on the demographic distribution of patients with different salivary gland carcinomas treated at a major regional Spanish hospital serving around 750,000 patients. Little information is available on the characteristics of salivary gland carcinomas patients in Spain. One study reported a low incidence of major salivary gland carcinomas in this country that tended to decrease between 1991 and 2001 (8) contrasting with reports of an increasing incidence in some other countries, including the USA (9). This discrepancy may be attributable to differences in behaviors and risk factors among populations.

The mean age of the 149 salivary gland carcinomas patients in the present study was 55.6 yrs, within the range of reports in the literature from 45.2 (10) to 58.6 yrs (11). The peak incidence was in the eighth decade, much older than observed in Asia and Nigeria (10-16) and older than the seventh decade reported in Brazil (6) and the fifth decade in Mexico (17). There was no gender difference in the present study, with a male:female ratio of 1, similar to findings by Luksic *et al.* (1), Wang *et al.* (5), and Tian *et al.* (15). However, various authors have found salivary gland carcinomas to be more frequent in females (11,13,18,19). Thus, a male:female ratio of 0.96 was reported in one of the longest series, derived from a review of cases in Sweden over a period of almost 30 years records (20) and a ratio of 0.42(7). In contrast, salivary gland carcinomas were found to be more frequent in males than in females by some other researchers (3,6,10,14,16,21).  Table 3 summarizes the histologic diagnoses reported in the literature.

**Table 3:**
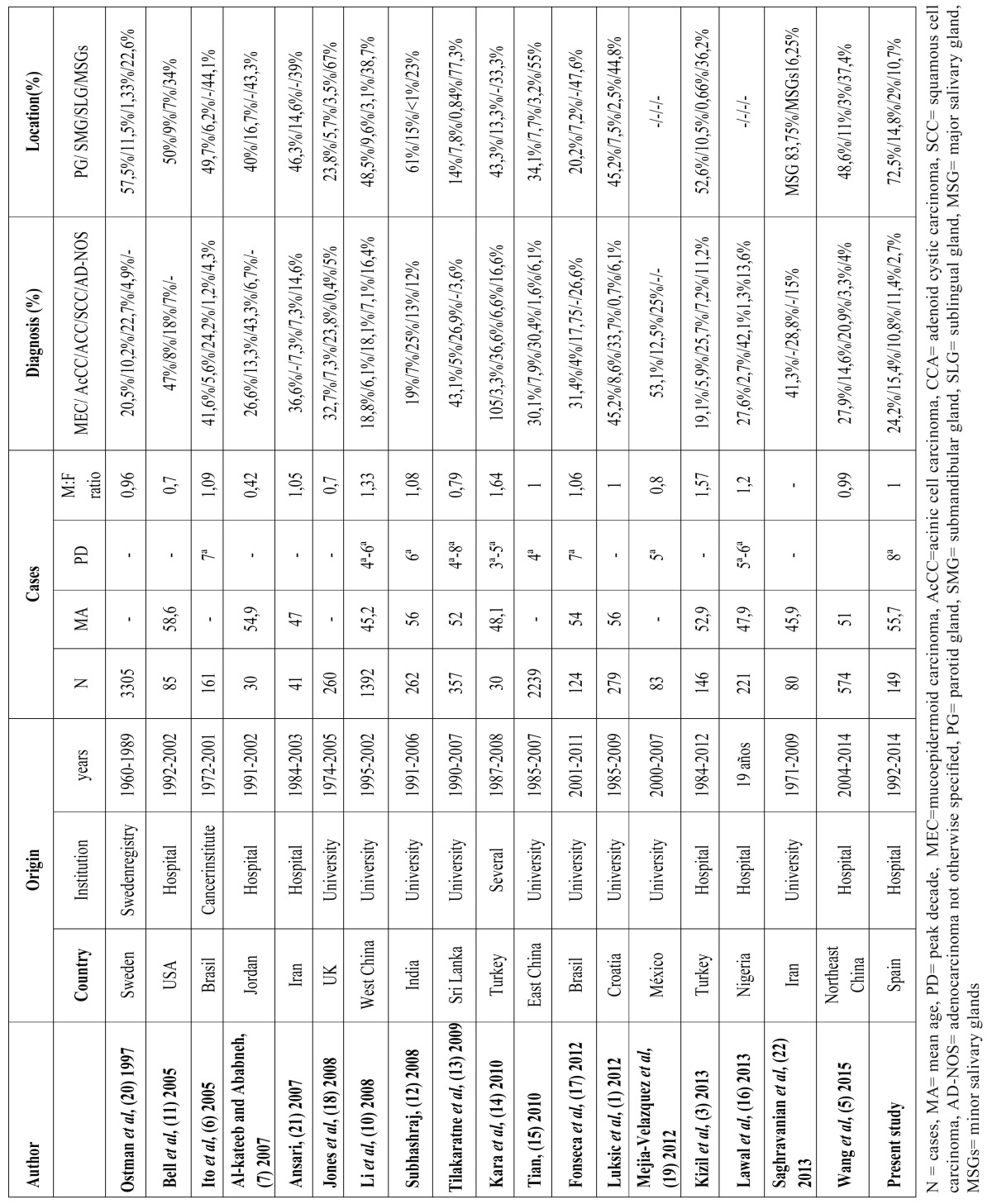
Comparison of studies on salivary gland carcinomas.

The parotid gland was the most frequent localization (72.5%) in our study, as in most large case series (3,5,10-12,14,20). However, minor salivary glands were the most frequent site in some other studies (7,13,15,18) and were the second most frequent in most reports (3,6,10,11,14,20,21). The type of center in which cases originate may affect this result, given that university centers are more likely to encounter minor salivary glands lesions than major salivary gland lesions, which are generally treated in hospital settings. In the present series, the submandibular gland was the second most frequent localization, followed by minor salivary glands, similar to the report by Saghravanian *et al.* (22), in which 16.25% of cases were minor salivary glands. It should be taken into account that the biopsy results are classified by anatomic site in our hospital and some minor salivary glands may be categorized as of intraoral rather than salivary gland origin, producing a possible underestimation. Only 2% of the salivary gland carcinomas were in the sublingual gland, consistent with previous findings of the very low prevalence (1,3,5,10,12,13,15,18,20) or absence of cases at this site (6,7,14,17,21), although a prevalence of 7% was reported in one study (11). Among the minor salivary glands, the most frequent salivary gland carcinomas localization was the palate, as also reported in other similar series (1,3,5,7,16).

MEC was the most frequent histopathological diagnosis (24.2%), as found by various authors (5,6,11,13,18,19,21,22) followed by AcCC (15.4%) and SCC (11.4%). In some other series, the percentages of MEC and ACC cases were similar (10,15) or ACC was the most frequent (3,7,5,12,14,16,20). It should be borne in mind that ACC frequently affects minor salivary glands and that PLGA was considered an ACC in the past (6). Since its first description in 1983, a relatively high frequency of PLGA has been reported in some studies (23). Both ACC and PLGA show a cribriform morphology and perineural infiltration; therefore, the differential diagnosis, which is highly critical for the management and prognosis of patients, can be difficult in a small biopsy (24). In our series, 6.1% of salivary gland carcinomas were PLGAs, similar to findings of Al-kateeb *et al.* (7), and they were all localized in minor salivary glands. A study by de Souza *et al.* (8) found differences in PLGA frequency between continents, ranging from 3.9% in Asia to 20% in Oceanía, consistent with data recently published by Wang *et al.* (5). Luksic ateeb and Ababneh (1,7) reported no cases of PLGA, which may in part explain why ACC was the most frequent diagnosis (43.3%). Kizil *et al.* (3) observed PLGA in 5.9% of cases and, although ACC was the most frequent, the percentage of cases was lower (25.7%). A higher percentage of SCC cases (11.4%) was recorded in the present series than in other studies (1,6,18,20), and one explanation may be the classification of high-grade MECs as SCCs or undetermined adenocarcinomas in some cases (13). In most studies, either MEC or ACC was the most frequent diagnosis. AcCC was the second most frequent diagnosis in our patients (15.4%), with a similar frequency to that reported in Mexico (19). According to the WHO in 2005 and Kizil *et al.* (3), the AcCC is slightly more common among females, and in our series, the male:female ratio was 0.44. PLGA was more frequent in females, as also reported by Jones *et al.* (18). According to other authors, ACC (4,14) and SCC (3) were more frequent in males. The mean ages at diagnosis in our series were consistent with the few published data on this variable, being around 50 years of age for ACC (3) and MEC (3,6) and higher (70.8 yrs) for SCC (5,18) .

In summary, the distribution of salivary gland carcinomas in this Spanish population, including the histological type and the age and sex of patients, was similar to previous reports in different countries ( Table 3) except for a higher frequency of AcCC and SCC cases. These results confirm the higher frequency of AcCC and PLGA in females and of SCC and ACC in males. Further epidemiological studies in European populations are warranted to improve understanding of salivary gland carcinomas and develop strategies for their early diagnosis and management.
